# The Impact of Probiotics and Prebiotics on Dry Eye Disease Signs and Symptoms

**DOI:** 10.3390/jcm11164889

**Published:** 2022-08-20

**Authors:** Azadeh Tavakoli, Maria Markoulli, Eric Papas, Judith Flanagan

**Affiliations:** 1 Brien Holden Vision Institute, Sydney 2052, Australia; 2 School of Optometry, Vision Science University of New South Wales, Sydney 2052, Australia; 3 Vision CRC, Sydney 2052, Australia

**Keywords:** dry eye disease, probiotic, prebiotic, eye symptoms, treatment, clinical research

## Abstract

Dry eye is considered an inflammatory disease. Gut microbiota are important in the regulation of low-grade chronic inflammation, including in the eye. Probiotics and prebiotics are increasingly used to regulate chronic-disease-associated gut dysbiosis. Therefore, this double-masked, randomized controlled clinical trial aimed to explore the potential of oral probiotics and prebiotics in the management of dry eye disease. In total, 41 participants with dry eye received probiotic and prebiotic supplements (treatment group, *n* = 23) or respective placebos (control group, *n* = 18) for 4 months. Dry eye symptoms and signs were evaluated using the Ocular Surface Disease Index (OSDI), Dry Eye Questionnaire 5, osmolarity, non-invasive keratograph break-up time (NIKBUT), ocular surface staining, tear meniscus height (TMH), lipid layer thickness, and conjunctival redness. After 4 months, the average OSDI score of the treatment group was significantly better compared to that of the controls (16.8 ± 5.9 vs. 23.4 ± 7.4; *p* < 0.001). The NIKBUT and TMH did not change significantly with treatment (*p* = 0.31 and *p* = 0.84) but reduced significantly for controls on average by −5.5 ± 1.0 secs (*p* = 0.03) and 0.2 ± 0.1 mm (*p* = 0.02). These data suggest that probiotics and prebiotics might be effective in the management of dry eye disease.

## 1. Introduction

Dry eye disease is considered one of the most common ocular surface diseases, with a global prevalence of 11.59%, depending on the chosen diagnostic criteria [1]. In 2017, the second TFOS DEWS report defined dry eye as a “…multifactorial disease of the ocular surface characterized by a loss of homeostasis of the tear film, and accompanied by ocular symptoms, in which tear film instability and hyperosmolarity, ocular surface inflammation and damage, and neurosensory abnormalities play etiological roles” [2]. This definition highlights the multifactorial etiology but also the use of “disease” suggests pathological outcomes that decrease of quality of life of patients [3]. Lack of homeostasis suggests that various perturbations of the ocular environment might trigger the disease [2,4,5].

Dry eye disease is an inflammatory condition that has many features in common with autoimmune disease [6]. Altered immunity is a significant factor in dry eye. As articulated by Stern and colleagues [7], dry eye disease is increasingly recognized as a localized autoimmune disease driven by dysregulated immunoregulatory and inflammatory pathways of the ocular surface. Mucosal tolerance disruption is integral to the pathogenesis of dry eye disease [8], initiated when the immune balance of the ocular surface is altered due to internal or external factors. Stress to the ocular surface initiates a cascade of acute response cytokines and sequestering of auto-response T cells that results in a chronic autoimmune response [7].

The gastrointestinal tract is inhabited by a vast number of microorganisms. The similar function of ocular surface mucins and glycoproteins to those in the gastrointestinal tract and the fact that the mucous membranes are connected throughout the body support the hypothesis that the gut microbiota can affect the health of different parts of the body, including the eye [9]. The gut microbiota, through the production of metabolites, mucosal mediators, and systemic immune responses, play an important role in the regulation of the immune system. An increasing number of studies have indicated alteration of the gut microbiota in Sjögren’s syndrome [10,11,12] and the correlation of gut dysbiosis with dry eye severity [13,14]. Reduced gut microbiota diversity in dry eye patients compared to the control group has also been found [15].

The modification of gut composition through the normalization of its microbiota is a solution for the treatment of gut dysbiosis and may pave the way for novel therapeutic approaches to treat and manage various diseases in different parts of the human body, including the eye [16]. There are three common methods for altering the gut microbiota. One is fecal microbiota transplantation, another is the application of probiotics (potentially beneficial microorganisms), and the third is the application of prebiotics (for boosting specific populations of microorganisms). The last one can be used with probiotics, the combination of which is referred to as “symbiotic”. Several small studies have provided evidence for the efficacy of this approach in the short term. Chisari et al., in 2016, found that a mixture of E. faecium LMG S-28935 and Saccharomyces boulardii MUCL 53837 decreased subjective symptoms with an increase in both tear secretion and tear break-up time [17]. Another pilot study by Chisari et al. reported that a 30-day supplementation of *B. lactis* and *B. bifido* significantly increased tear secretion and tear break-up time compared to placebo, in addition to alteration of the ocular microbiota, in 20 dry eye patients [18]. Similarly, Kawashima et al. noted that the consumption of *E. faecium* WB2000 mixed with fish oil for 8 weeks improved subjective symptoms, with increased tear secretion, in dry eye patients [19].

Although these reports are promising, this is a relatively novel field of research and the number and duration of studies have, so far, been limited. More comprehensive investigations are needed that can help inform clinicians about the practical application of such treatments. Therefore, the current hypothesis is that the administration of factors, such as probiotics, prebiotics, or symbiotic combinations, that can regulate the function of the gut microbiota can improve the outcomes (symptoms and/or signs) of dry eye disease. Consequently, this double-masked, randomized, controlled longitudinal trial aimed to explore the potential of oral probiotics and prebiotics in reducing the severity of signs and symptoms of dry eye disease through systemic and localized (ocular) immune function modulation.

## 2. Materials and Methods

Participants were included if DEQ-5 ≥ 6 or the OSDI ≥ 13 plus at least one of the following was present: non-invasive tear break-up time <10 s and ocular surface staining (>5 corneal spots or >9 conjunctival spots, lid margin ≥2 mm length, and ≥25% width) [20]. Participants with known dry eye disease were recruited from the databases of the Brien Holden Vision Institute, the UNSW Optometry Clinic, staff, and students at the UNSW School of Optometry and Vision Science. Participants included in the study were healthy, with no ocular and systemic inflammatory and autoimmune disorders. All participants were aged 18 years and above. Participants were excluded if they were taking probiotic/prebiotic commercial supplements. Participants were advised not to change their diet for the duration of the study. Exclusion criteria also included any systemic or topical medications that affect ocular physiology or the tear film, e.g., anti-acne medications (such as Roaccutane) and corticosteroids or immunosuppressant medications (such as Hydrocortisone and Prednisolone). Participants did not enroll in the study if they had undertaken ocular surgery within 12 weeks and corneal refractive surgery within 3 years prior to enrolment for this trial. Furthermore, participants were excluded if they were taking oral or topical antibiotics. Participants were asked to not wear their lenses on the day of study visits if they were contact lens wearers. Participants with ocular injury and active corneal infection or any active ocular disease were excluded from the study. Pregnant or lactating women were excluded from the study.

This was a double-masked, randomized, controlled clinical trial in which a total of 41 subjects with mild to severe dry eye were enrolled and randomized through a web-based system into two groups, treatment and control. The treatment group received probiotic supplements (in the form of capsules) and prebiotic supplements (in the form of sachets). The control group received a probiotic placebo (in the form of capsules) and a prebiotic placebo (in the form of sachets). The treatment duration was 4 months, and the participants were followed up at 1 month and 4 months after commencing treatment and again 1 month after treatment cessation.

MULTIBIOTIC™ Probiotics (Medlab Pty Ltd., Botany, NSW, Australia) and maltodextrin (placebo) in the shape of hard capsules were used. Sachets of NutriKane D (MediKane Pty Ltd., Macquarie Park, NSW, Australia) and maltodextrin were used as prebiotics and the prebiotic placebo, respectively. MULTIBIOTIC™ probiotic contains 21.075 billion CFU of bacteria per capsule, including *Streptococcus*, *Lactobacillus*, and *Bifidobacterium* species. NutriKane D contains Phytocell (Kfibre) and red sorghum flour. Previous studies have investigated the efficacy of these supplements in altering the gut microbiota and reducing systemic inflammation in the body [21,22]. 

All measurement were performed by one investigator (AT), who was masked regarding the allocation of interventions to participants. 

**Dry eye symptoms:** Ocular symptoms were assessed by the administration of the Ocular Surface Disease Index (OSDI) and Dry Eye Questionnaire 5 (DEQ-5) [20]. Both questionnaires were included in this study to provide a better understanding of ocular symptoms. 

**Ocular surface health and staining:** Slit-lamp biomicroscopy was used to check ocular health and integrity. Corneal staining was evaluated using sodium fluorescein (OptiStrips-FL). Conjunctival and lid margin staining was assessed using lissamine green (Green Glo). Corneal staining was evaluated under cobalt blue light, while conjunctival and lid staining was assessed under white light. Corneal and conjunctival staining was graded according to Sjögren’s International Collaborative Clinical Alliance (SICCA) ocular staining score [23]. The sum of the staining in both eyes was analyzed. Eyelid staining was scored according to the modified grading scale by Korb et al. [24].

**Tear film osmolarity:** Tear film osmolarity was assessed using the I-PEN osmolarity system (I-MED Pharma Inc., Dollard-des-Ormeaux, QC, Canada, (https://imedpharma.com/, accessed on 20 June 2022). This is a portable battery-operated unit that consists of a handheld unit with a display screen to show the osmolarity test results and a single-use disposable card that comes in contact with the tear film. Osmolarity measurement for each eye was conducted as per the I-PEN manufacturer’s instructions [25]. The repeatability of this device has been previously tested and reported [26]. 

**Non-invasive keratograph break-up time(NIKBUT):** Tear film stability was assessed automatically using the Oculus Keratograph (Oculus, Wetzlar, Germany). The participants were instructed to blink naturally two times and then to cease blinking until instructed to blink again. Three measurements were performed for each eye and the average for each eye included for analysis. 

**Meniscometry:** The tear meniscus assessment height (TMH) was measured with the Oculus Keratograph (Oculus, Wetzlar, Germany).

**Tear Lipid Layer assessment:** The thickness of the lipid layer of the tear film was assessed with the LipiView interferometer (TearScience, Morrisville, NC, USA). 

**Ocular Redness Assessment:** The presence of conjunctival redness can indicate ocular surface inflammation [27,28,29]. The quantitative assessment of bulbar conjunctival redness was performed with the Oculus Keratograph (Oculus, Wetzlar, Germany).

The order of the measurements was from least invasive to most invasive, as follows: tear lipid layer assessment, TMH, NIKBUT, ocular redness assessment, osmolarity, and ocular staining. There was a 5 to 10 min gap between each measurement. The measurements were conducted in the same examination rooms with a stable temperature (20 ± 3 °C) for all participants. 

The data were first entered into Microsoft Excel (Microsoft Corp., Redmond, WA, USA) and then exported to IBM SPSS Statistics version 26.0 for statistical analysis (IBM Corp., Armonk, NY, USA). A generalized linear model was used to investigate changes over the study visit timelines. Differences between time points were checked with non-parametric and paired *t*-tests where the model indicated significance. Confidence intervals were set at 95%, and a *p*-value below 0.05 was used as an indicator of statistical significance.

## 3. Results

In total, 41 participants were recruited in this study, of which 32 completed the treatment period. Participants were aged 18 years and above (18–76), with a mean age of 41 ± 16 years. Among them, 30 were female and 11 were male. Interventions were given to the participants in a random order, resulting in 23 participants receiving treatment supplements and 18 receiving the placebo. The average age in the treatment and control groups were 41 ± 16 years and 41 ± 17 years, respectively. There were 14 females and 16 males in the treatment group and 9 females and 2 males in the control group. 

### 3.1. Dry Eye Symptoms

**Figure 1:**
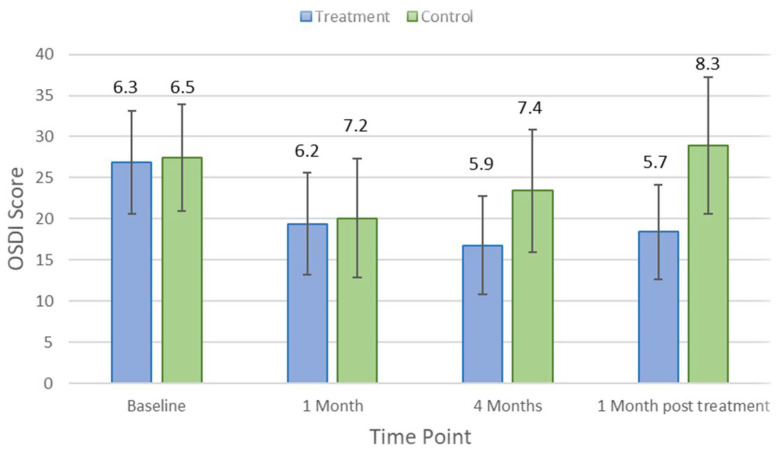
Mean Ocular Surface Disease Index (OSDI) score at each study time point in the treatment and control groups. Error bars are 95% confidence intervals.

There were no significant differences in comfort scores between control and treatment groups at baseline (*p* > 0.05). Figure 1 shows the changes in the Ocular Surface Disease Index (OSDI) score over time in the treatment and control groups. At the first-month visit, the ODSI score improved in both treatment (*p* = 0.03) and control (*p* = 0.02) groups. After 4 months of treatment from the baseline visit, the average OSDI score in the treatment group was significantly better than that in the control group (16.8 ± 5.9 vs. 23.4 ± 7.4, respectively; *p* < 0.001). At the follow-up visit, which occurred 1 month after treatment cessation, the average OSDI score in the control group was significantly worse than that in the treatment group (28.9 ± 12.7 vs. 18.4 ± 12.7, respectively; *p* = 0.03). 

After 1 month, the DEQ-5 score improved significantly in the control group (*p* = 0.03) but did not change in the treatment group (*p* = 0.08). After the treatment period, the DEQ-5 score did not change significantly in either the treatment group (8.8 ± 4.1; *p* = 0.06) or the control group (9.6 ± 3.2; *p* = 0.40). Changes in OSDI and DEQ-5 scores from the baseline were not influenced by sex (*p* > 0.05) nor did they correlate with age at any time point in either the test group or the control group (*p* > 0.33).

### 3.2. Dry Eye Signs

**Figure 2:**
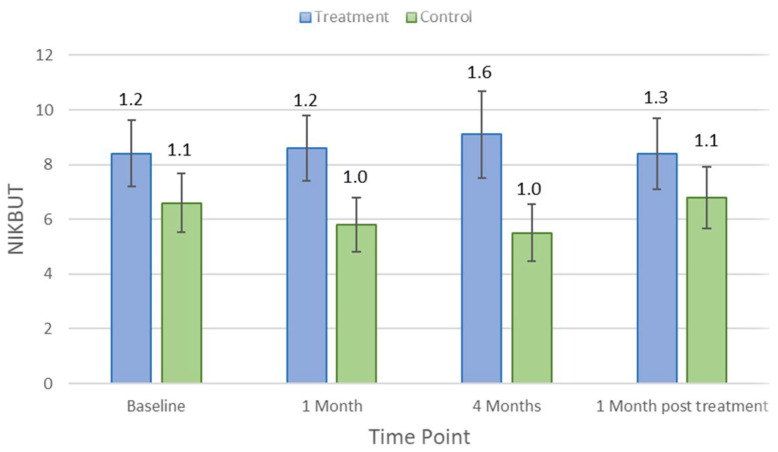
Mean non-invasive keratograph break-up time (NIKBUT) at each study time point in the treatment and control groups. Error bars are 95% confidence intervals.

**Figure 3:**
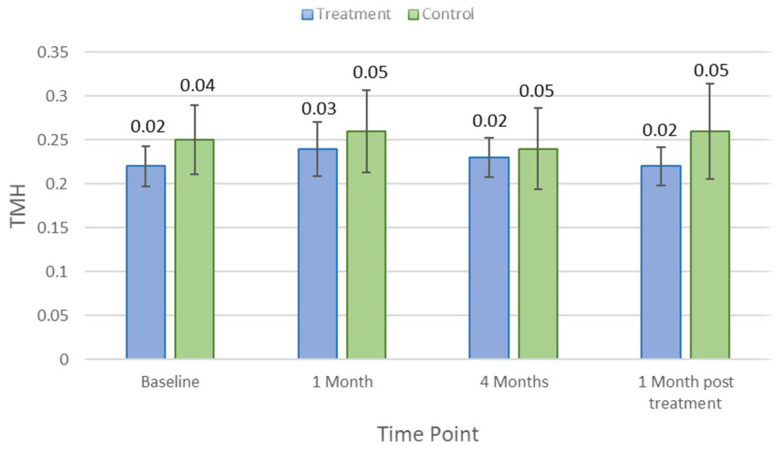
Mean meniscus assessment height (TMH) at each study time point in the treatment and control groups. Error bars are 95% confidence intervals.

Table 1 indicates the average and *p*-values for each clinical parameter over different study visit timelines. There was no significant difference in clinical measures between control and treatment groups at baseline (*p* > 0.05). Lipid layer thickness did not change significantly in the treatment group (*p* = 0.18) but reduced by an average of 8.5 ± 5.7 nm (*p* = 0.03) in controls after 1 month of the treatment. There were no significant changes in other clinical parameters, including TMH, NIKBUT, tear osmolarity, ocular staining, conjunctival bulbar redness, and meibomian gland secretion (*p* > 0.05), at the first-month visit in either the treatment group or the control group. After the 4-month treatment period, NIKBUT and TMH did not change significantly for the treatment group (*p* = 0.31 and *p* = 0.84) but reduced significantly for controls by an average of −5.5 ± 1.0 s (*p* = 0.03) and 0.2 ± 0.1 mm (*p* = 0.02), respectively. Figure 2 and Figure 3 show how NIKBUT and TMH changed over time in the treatment and control groups. There were no significant changes in either the treatment group or the control group for other clinical parameters, including lipid layer thickness, tear film osmolarity, conjunctival redness, and ocular staining, at each visit (*p* > 0.05). 

At the first-month visit, one participant in the treatment group was found to no longer satisfy the criteria for dry eye and at 4 months, a further four participants in the treatment group were no longer dry eye positive. At the follow-up visit, this number was reduced to one participant in the treatment group. No one in the control group converted out of the dry eye diagnosis at any of the study time points.

## 4. Discussion

The results of this study indicate that regular consumption of probiotics and prebiotics can reduce dry eye symptoms, as assessed by the OSDI. Furthermore, taking these supplements may improve tear secretion and stability, therefore stabilizing some clinical signs, including tear break-up time and tear meniscus height, over time. Modulating the gut has been shown to reduce systemic inflammation. Given the connection between the ocular and gut mucosa, we hypothesize that ocular surface inflammation will also be improved, thereby reducing the signs and symptoms of dry eye. Modulating the gut microbiome has been shown to also modulate the proteins expressed by the lacrimal glands, with IL-10 increasing and IL-1β and IL-6 decreasing [30]. Therefore, the stability of some clinical features in this study, including TMH and NIKBUT, might be due to the effect of probiotics in changing the expression of inflammatory markers associated with immunomodulation in lacrimal glands.

During the present study, tear film stability in the control group reduced after 4 months, but no change was seen for the treatment group over the same period. Dry eye disease is a multifactorial disease, and environmental factors are significant contributors to this disease. As such, the observed changes in the control group may have been due to the conditions prevailing at the time. For example, the data collection for this study was conducted mostly during the COVID-19 pandemic, when the widespread use of face masks was required to prevent the spread of disease. A marked increase in dry eye symptoms among regular mask users has been reported [31,32,33], which can manifest as increased ocular irritation and reduced tear break-up time [34]. Moreover, fewer social interactions as a result of the pandemic contributed to increased computer time, which could contribute to evaporative-type dry eye disease [35]. The factors may have precipitated the increased dry-eye-type responses among controls; however, the data suggest that probiotic and prebiotic treatment can mitigate the effect of environmental factors on dry eye.

In this study, the dry eye symptoms improved in both groups after 1 month of taking the interventions. This could be because of the placebo effect [36]. Nevertheless, this effect waned over the course of the study, and the treatment group showed better improvement in their symptoms after the full-term treatment period. In contrast to symptoms, the clinical features did not change after 1 month of taking the intervention for either group. This can indicate a longer time required for these supplements to change the gut composition and to be reflected in the clinical signs of dry eye disease.

This study did not find an improvement in corneal, conjunctival, and lid wiper staining scores, potentially because participants did not have severe ocular staining from the outset. Moreover, people with autoimmune diseases such as Sjögren’s syndrome were excluded from this study. It is possible that more substantial changes in dry eye signs and symptoms after treatment with probiotics and prebiotics could be observed in patients with Sjögren’s syndrome as their gut microbiota are more significantly different compared to those with environmental dry eye or the healthy cohort, with a concomitantly higher level of gut dysbiosis [13,37]. Choi et al. reported reduced levels of ocular surface inflammation using probiotics in a mouse model of autoimmune dry eye. They found a lower ocular staining score and higher tear secretion in mice treated with probiotics [30]. However, the safety of probiotic use in human autoimmune disease is a matter of discussion because *Lactobacillus* spp. has been reported to act as a possible pathogen [38]. Considering the promising evidence of the beneficial impact of probiotics on the dry symptoms and clinical signs, future clinical studies are necessary to further investigate probiotics’ benefits for patients with Sjögren’s syndrome.

The gut microbiota vary according to non-modifiable factors, such as ethnicity and gender, as well as being modifiable by diet [16]. In this study, participants were asked not to change their diet during their enrolment, but as this is difficult to control, there may have been some residual impact on the outcomes.

New evidence is emerging on the role of the gut microbiota in inflammatory ocular disease [39,40], yet investigations into the effect of probiotics and/or prebiotics on dry eye disease are still in the early stages. Thus, there is currently no guidance regarding the proper dosage, duration, and formulation of these supplements. It is possible, therefore, that stronger effects than those observed here may be associated with alternative dosing regimens.

## 5. Conclusions

This study showed that the application of probiotics and prebiotics might be effective in the management of dry eye disease and suggests a potential alternative therapeutic treatment for dry eye disease management. Future investigations are necessary to establish customized probiotic and/or prebiotic interventions with an optimized modulation of the gut microbiota to treat dry eye disease.

## Figures and Tables

**Table 1:** Summary statistics for clinical variables in the treatment and control groups at each time point. *p*-Values indicate a comparison between the time point and the baseline.

Variable	Baseline		1st Month		4th Month		1 Month Post Treatment	
	Control	Treatment	Control	Treatment	Control	Treatment	Control	Treatment
OSDI (0–100)	27.4 ± 14.0	26.9 ± 15.3	20.1 ± 14.7, **0.02**	19.4 ± 14.5, **0.03**	23.4 ± 13.7, 0.05	16.8 ± 13.2, **0.01**	28.9 ± 12.7, 0.03	18.4 ± 12.7, 0.22
DEQ-5 (0–22)	10.2 ± 3.1	11.0 ± 3.4	8.8 ± 3.1, **0.03**	9.7 ± 3.7,0.08	9.6 ± 3.3, 0.40	8.8 ± 4.1, 0.06	8.7 ± 3.2, 0.41	10.1 ± 3.4, 0.35
LLT (nm)	57.4 ± 17.2	72.6 ± 21.8	48.9 ± 15.9, **0.04**	67.2 ± 23.3, 0.18	60.3 ± 23.4, 0.40	70.3 ± 18.9, 0.80	55.7 ± 17.4, 0.22	71.8 ± 22.6, 0.89
TMH (mm)	0.3 ± 0.1	0.2 ± 0.1	0.3 ± 0.1,0.60	0.2 ± 0.1,0.30	0.2 ± 0.1, **0.02**	0.2 ± 0.1, 0.84	0.3 ± 0.1, 0.60	0.3 ± 0.1, 0.75
NIKBUT (s)	6.7 ± 3.3	8.4 ± 4.2	5.8 ± 2.9, 0.20	8.6 ± 3.9,0.90	5.5 ± 2.7, **0.03**	9.1 ± 5.0, 0.30	6.8 ± 2.7, 0.67	8.4 ± 4.2, 0.44
TO (mOsm/l)	298.9 ± 19.8	299.7 ± 14.6	301.4 ± 14.8, 0.60	301.4 ± 3.6, 0.63	304.3 ± 16.5, 0.22	301.8 ± 14.5, 0.51	309.1 ± 12.1, 0.60	297.8 ± 14.0, 0.75
CCS	0.7 ± 1.1	0.9 ± 0.8	0.9 ± 1.2, 0.50	0.8 ± 0.9, 0.80	0.7 ± 0.8, 0.94	0.8 ± 0.9, 0.85	0.6 ± 0.7, 0.60	0.8 ± 0.9, 0.76
LS	0.8 ± 1.0	0.7 ± 0.9	1.0 ± 1.0, 0.60	0.6 ± 0.8, 0.90	0.8 ± 0.7, 0.56	0.6 ± 0.9, 0.76	0.8 ± 0.9, 0.45	0.8 ± 0.9, 0.37
BR (mm^2^)	0.9 ± 0.5	1.0 ± 0.5	0.9 ± 0.6, 0.80	0.9 ± 0.5, 0.14	0.9 ± 0.6, 0.40	0.9 ± 0.4, 0.91	1.0 ± 0.5, 0.10	1.0 ± 0.5, 0.11
MGS	2.7 ± 0.8	2.4 ± 1.1	2.9 ± 0.3, 0.13	2.6 ± 0.8, 0.32	2.8 ± 0.7, 0.98	2.5 ± 1.0, 0.56	2.8 ± 0.4, 0.40	2.3 ± 1.0, 0.14

OSDI: Ocular Surface Disease Index; DEQ-5: Dry Eye Questionnaire 5; LLT: lipid layer thickness; TMH: tear meniscus height; NIKBUT: non-invasive keratograph break-up time; TO: tear osmolarity; CCS: corneal and conjunctival staining; LS: lid staining; BR: bulbar redness; MGS: meibomian gland secretion. In this table, the first and second numbers refer to the mean ± standard deviation, respectively, and the third numbers refer to the *p*-value relative to baseline. The numbers in bold are statistical significance *p*-values.

## Data Availability

The data presented in this study are available on request from the corresponding author. The data are not publicly available due to ethical reasons.
